# Physical inactivity is associated with chronic musculoskeletal complaints 11 years later: results from the Nord-Trøndelag Health Study

**DOI:** 10.1186/1471-2474-9-159

**Published:** 2008-12-01

**Authors:** Helene Sulutvedt Holth, Hanne Kine Buchardt Werpen, John-Anker Zwart, Knut Hagen

**Affiliations:** 1 Department of Neuroscience, Faculty of Medicine, Norwegian University of Science and Technology, Trondheim, Norway; 2 Norwegian National Headache Centre, section of Neurology, St. Olav's Hospital, Trondheim, Norway; 3 Department of Neurology, Ullevål University Hospital, Oslo, Norway; 4 Faculty of Medicine, University of Oslo, Oslo, Norway

## Abstract

**Background:**

Physical inactivity is associated with several diseases, but studies evaluating the association between chronic musculoskeletal complaints (MSCs) and physical exercise have shown conflicting results. The aim of this large-scale prospective population-based study was to investigate the association between self-reported physical exercise at baseline and the prevalence of chronic musculoskeletal complaints (MSCs) 11 years later.

**Methods:**

The results are based upon two consecutive public health studies conducted within the county of Nord-Trøndelag, Norway (The HUNT studies). A total of 39,520 (83%) out of 47,556 adults who participated in HUNT 1 and HUNT 2 responded to questions about physical exercise at baseline in 1984–86, and to questions about musculoskeletal complaints 11 years later (1995–97). Chronic MSCs was defined as MSCs ≥ 3 months during the past year, and chronic widespread MSCs such as pain ≥ 15 days during the last month from the axial region, above the waist, and below the waist. Associations were assessed using multiple logistic regression, estimating prevalence odds ratio (OR) with 95% confidence intervals (CIs). All the final analyses were adjusted for age, gender, body mass index, smoking and education level.

**Results:**

At follow-up 20,223 (51%) reported chronic MSCs, and among these 2,318 (5.9%) reported chronic widespread MSCs. Individuals who exercised at baseline were less likely to report chronic MSCs 11 years later (OR 0.91, 95% CI 0.85–0.97) than inactive persons. Among individuals who exercised more than three times per week, chronic widespread MSCs were 28% less common (OR 0.72, 95% CI 0.59–0.88) compared to inactive individuals.

**Conclusion:**

In this large-scale population-based study, physical exercise was associated with lower prevalence of chronic MSCs, in particular chronic widespread MSCs. Future studies should try to clarify whether chronic MSCs are a cause or a consequence of inactivity.

## Background

Chronic musculoskeletal complaints (MSCs) are among the major health problems in Western society, and the most frequent cause of long-term sickness leave in Norway [1,2]. In recognition of this, the WHO has endorsed the Bone and Joint Decade 2000–2010, seeking more knowledge about the epidemiology of chronic MSCs [1]. Chronic MSCs is associated with several negative determinants of health, such as smoking, overweight, and low socio-economic status [3-6]. Increased risk of cancer [7] and higher mortality [8] have also been reported among individuals with chronic widespread MSCs, which further emphasizes that this group of patients may constitute an important public health problem.

Physical inactivity is, like chronic MSCs, associated with e.g. more sick leave, overweight, low socio-economic status, increased risk of cancer, and increased mortality [9-14]. Although chronic MSCs and physical inactivity share several negative determinants of health, previous studies have shown inconsistent results regarding the influence of physical exercise on MSCs [15]. In some cross-sectional studies, physical inactivity has been associated with high prevalence of MSCs [16-19]. In a 14 year prospective study of older adults, long term physical activity was associated with about 25% less MSCs compared to sedentary controls [20]. In contrast, vigorous exercise did not prevent development of widespread pain in children [21]. Furthermore, physical activity was not listed among the most consistently-associated factors with MSCs in a review of epidemiological studies [22]. These inconsistent findings may be due to lack of, or vague, definition of the level of physical activity [22,23]. Other possible reasons are the fact that most previous studies evaluating the association between physical activity and MSCs have been cross-sectional with a relatively low number of participants, and that previous studies often have focused upon a defined subgroup of the population [16-20,24].

The aim of this large-scale population-based study was to investigate the association between self-reported physical exercise, and the prevalence of chronic MSCs 11 years later.

## Methods

Two extensive public health surveys have been performed in the county of Nord-Trøndelag, Norway (The Nord-Trøndelag Health Studies [HUNT]). All inhabitants 20 years and older were invited to participate in both studies, which included questionnaires and physical examination e.g. measurement of blood pressure, body weight and height. Details of these two comprehensive health studies, covering a wide range of topics, are described elsewhere [25,26].

In HUNT 1 (1984–86), 74,599 (88%) of 85,100 eligible individuals answered the questionnaire that was sent with the invitation, and also participated in the health examination. A detailed description of the study population, analysing both participants and non-participants, has been published previously [25]. In HUNT 1, no questions about musculoskeletal symptoms were included.

In HUNT 2 (1995–97), 66,140 (71%) of 92,936 individuals participated [25]. A total of 47,556 individuals, representing 72% of all persons included in HUNT-2, participated in both surveys (HUNT 1 and HUNT 2) [26]. Among these, 39,520 individuals (83%) responded to the questions about physical exercise in HUNT 1 and to the questions about MSCs in HUNT 2. Of these, a total of 34,482 persons (87%) also reported their level of physical exercise.

This study was approved by the Regional Committee for Ethics in Medical Research, and the HUNT study was also approved by the Norwegian Data Inspectorate.

### Physical exercise

In HUNT 1 the participants were asked to report their weekly average of leisure-time physical exercise (that is, walking, skiing, swimming, or other sports without further specification) with five response alternatives (never, <1, 1, 2–3, ≥ 4 times). In the most simple classification (i.e. classification 1) individuals who reported "never" were defined as "inactive", the remaining "active." In classification 2 the active individuals were divided into four separate categories based on frequency of exercise per week.

Participants who exercised once a week or more were also asked about the average duration (<15, 15–30, 30–60, >60 min) and intensity (light, moderate, vigorous) of the exercise. The questions related to duration and intensity have been validated against measured oxygen uptake (VO_2 max_) and heart rate (HF_max_), and provide a useful measure of leisure-time physical exercise [27]. In the present study we also constructed a summary score of frequency, duration, and intensity which has been used in several other studies (i.e. classification 3) [10,12,28]. The following indicate scores for each response of questions 1 and 3 were used were when calculating the summary score:

*a) Question 1 *(How often du you exercise?): never = 0; less than once a week = 0; once a week = 1; 2 to 3 times a week = 2.5; nearly every day = 7.

*b) Question 3 *(How long do you exercise each time?): less than 15 minutes = 0 minutes; 15–30 minutes = 23 minutes; 30 minutes to 1 hour = 45 minutes; more than 1 hour = 60 minutes.

Minutes per week of exercise were calculated and combined with the answers to *question 2 *(How hard do you exercise?): a) I take it easy without loosing my breath or braking into sweat=*light *physical activity; b) I push until I lose my breath and break into sweat = *hard *physical activity; c) I practically exhaust myself = *hard *physical activity.

Based on this summary score, the participants were divided into five separate categories (i.e. classification 3): *Inactive *= Participants answering *never *to question 1; *Very low*= 0–420 min/week *light *physical activity, or 0–59 min/week *hard *physical activity (except participants answering *never *to question 1);*Low *= 60–119 min/week, *hard *physical activity; medium = 120–179 min/week, *hard *physical activity; *High *= 180–420 min/week, *hard *physical activity

There were 84 individuals who did not respond to question 1. Based on their answers to the two other questions, they were placed either in the *active *group in classification 1, or the *very low *group in classification 3, but listed as *missing *in classification 2.

In HUNT 2 the participants were asked to report their weekly average duration of physical exercise with "light activity" (defined as no sweating or being out of breath) and "hard physical activity" (defined as sweating/being out of breath), with four response choices (0, <1, 1–2, ≥ 3 hours). Based on the duration of exercise per week, the participants were divided into five separate categories: inactive (0 hours), very low (<3 hours light activity), low (≥ 3 hours light activity), medium (1–2 hours hard activity), and high (≥ 3 hours hard activity). The validity and reliability of these questions have been evaluated, and the "hard" activity was a useful measure of vigorous physical exercise, whereas the "light" activity question had less reproducibility [29].

Classification 3 in HUNT 1 (Table 1) was used when evaluating the influence of change in activity level from HUNT 1 to HUNT 2. The category "decreased level" included individuals with a lower activity level in HUNT 2 than HUNT 1, whereas the group with "increased level" had a higher activity level in HUNT 2 than HUNT 1.

**Table 1:** Background data on individuals without and with chronic musculoskeletal complaints (subdivided to chronic widespread and non-widespread MSCs)

Variables	No chronic MSCs	Chronic MSCs	Chronic widespread MSCs	Chronic non-widespread MSCs
n = 39,520	19,297	20,223	2,318	17,905
Gender, female (%)	48.9	57.4	56.8	57.5
Mean age (SD)	55.6 (14.8)	57.7 (13.9)	55.3 (12.6)	58.0 (14.0)
Years of education (>= 13 years) (%)	12.6	8.7	8.8	8.7
Mean BMI (SD)	24.7 (3.5)	25.2 (3.8)	25.2 (3.9)	25.2 (3.8)
Current smoking (%)	30.4	34.7	38.4	34.2

### Musculoskeletal complaints

Both questionnaire 1 and 2 (Q1 and Q2) included questions about musculoskeletal symptoms adopted from the Standardized Nordic Questionnaire, which has previously been evaluated and found to give reliable estimates for low back and upper limb and neck discomfort, in particular for symptoms during the past year [30-32]. In Q1 participants were asked whether they had suffered pain or stiffness in muscles and joints lasting at least 3 months during the last year, whereas in Q2 they were asked to indicate the number of days during the last month with such complaints. In both Q1 and Q2 those who responded positively were then asked to indicate one or several of the following nine areas of the body: neck, shoulders, elbows, wrist/hands, upper back, low back, hips, knees and/or ankles/feet.

In the present study individuals with chronic MSCs (pain and/or stiffness ≥ 3 months during the past year) were subdivided into chronic widespread MSCs and chronic non-widespread MSCs. "Chronic widespread MSCs" were defined as pain and/or stiffness ≥ 3 month during the past year and ≥ 15 days with symptoms during the last month from all of the following regions: axial skeletal pain (pain in the neck, chest/abdomen, upper back, or lower back), pain above the waist (neck, shoulders, elbows, wrist/hands, chest/abdomen, or upper back) and below the waist (lower back, hips, knees, or ankles/feet). Individuals with chronic MSCs not fulfilling the criteria for chronic widespread MSCs were defined as having chronic non-widespread MSCs (mutually exclusive).

### Statistics

Baseline data were compared between individuals with or without chronic MSCs with independent sample t-test for continuous variables and with the chi-squared test for categorical variables. Two-tailed estimations of significance were used, and the level of significance was set at p < 0.05.

In the multivariate analyses, using logistic regression, we estimated the prevalence odds ratio (OR) with 95% confidence interval (CI) for the association between chronic MSCs (dependent variable) and physical exercise. Potential confounding factors were included in the multiple logistic regression analyses separately or together, but were excluded from the final analyses if the OR changed less than 0.05. Potential interaction between two variables was evaluated by including the product of the variables in the logistic regression analyses, and the interaction coefficient was tested using Wald χ^2 ^statistics. These confounders were age (5 year categories), gender, education (3 categories: ≤ 9, 10–12, ≥ 13 school years), body mass index (BMI, categorized into quartiles), smoking, alcohol consumption, blood pressure, and self assessed health. Age, BMI, education, smoking, and level of education stood out as important confounders, and therefore, all final analyses were adjusted for these. Data analyses were performed with the Statistical Package for the Social Sciences, version 15.0 (SPSS, Chicago, Illinois, USA).

## Results

Among the 39,520 participants, 20, 223 (51%) reported chronic MSCs, whereof 2,318 (5.9%) had chronic widespread MSCs and the remaining 17,905 (45.1%) chronic non-widespread MSCs. Individuals with chronic MSCs had significantly (p < 0.001) higher BMI and age, were more likely to be women and current smokers, and had a lower level of education compared to those without chronic MSCs (Table 1).

The prevalence of chronic MSCs were lower (OR = 0.91, 95% CI 0.85–0.97, Wald = 8.0, p = 0.005) among active than among inactive individuals. When considering frequency of exercise, individuals who performed physical exercise more than once a week had approximately 20% lower prevalence of chronic widespread MSCs compared to inactive persons (Table 2). A similar tendency was found for both genders; in males, chronic widespread MSCs were 25% less likely (OR 0.75, 95% CI 0.60–0.92, Wald = 7.5, p = 0.006) among those who exercised once a week or more compared to inactive individuals, whereas the corresponding OR for women was 0.82 (95% CI 0.67–1.00, Wald = 3.7, p = 0.055) (data not shown). Overall, chronic widespread MSCs were 28% less likely (0.72, 95% CI 0.59–0.88, Wald = 10.2, p = 0.001) among individuals who exercised more than three times per week compared to inactive persons (Table 2).

**Table 2:** Prevalence OR^#^of chronic musculoskeletal complaints (subdivided to chronic widespread and non-widespread MSCs) related to level of physical activity in HUNT 1

Activity level	Total	Chronic MSCs		Chronic widespread MSCs		Chronic non-widespread MSCs	
	39520	n	OR (CI)	n	OR (CI)	n	OR (CI)
**Classification 1**							
Inactive	4089	2239	1.0 (ref.)	261	1.0 (ref.)	1978	1.0 (ref.)
Active	35431	17984	0.9 (0.9–1.0)	2053	0.9 (0.8–1.0)	15927	0.9 (0.9–1.0)
**Classification 2**							
Inactive	4089	2239	1.0 (ref.)	261	1.0 (ref.)	1978	1.0 (ref.)
<1 times/week	11468	6100	1.0 (0.9–1.1)	791	1.0 (0.9–1.2)	5309	1.0 (0.9–1.1)
1 times/week	10590	5245	0.9 (0.8–0.9)	595	0.8 (0.7–0.9)	4650	0.9 (0.8–0.9)
2–3 times/week	8970	4452	0.9 (0.8–1.0)	474	0.8 (0.7–0.9)	3978	0.9 (0.9–1.0)
>3 times/week	4319	2140	0.9 (0.8–0.9)	193	0.7 (0.6–0.9)	1947	0.9 (0.8–0.9)
Missing	84	47	1.0 (0.7–1.6)	4	1.0 (0.3–2.8)	43	1.0 (0.7–1.6)
**Classification 3**							
Inactive	4089	2239	1.0 (ref.)	261	1.0 (ref.)	1978	1.0 (ref.)
Very low	29215	15114	0.9 (0.9–1.0)	1732	0.9 (0.8–1.0)	13382	0.9 (0.9–1.0)
Low	3664	1719	0.9 (0.8–1.0)	207	0.8 (0.7–1.0)	1512	0.9 (0.8–1.0)
Medium	1493	680	0.9 (0.8–1.1)	59	0.7 (0.6–0.9)	621	1.0 (0.9–1.1)
High	1059	471	0.8 (0.7–1.0)	59	0.9 (0.7–1.2)	412	0.8 (0.7–0.9)

^#^Adjusted for age, gender, body mass index, smoking and education.

When considering the activity level as a summary index of weekly physical exercise based on the combination of frequency, duration and intensity (classification 3), active participants were less likely to have chronic MSCs than those who were inactive (Table 2). Chronic widespread MSCs were 35% less likely (OR 0.65, 95% CI 0.48–0.87, Wald = 8.1, p = 0.004) among individuals with a medium activity level compared to inactive persons (Table 2).

Active individuals in HUNT 1 and HUNT 2 had a lower prevalence of chronic MSCs compared to inactive persons in both surveys (Table 3). A total of 29,913 (87%) out of the 34,482 who answered the questions about physical activity in both the surveys had maintained their activity level, whereas the remaining 4,569 (13%) had increased or decreased it. Increased activity level from HUNT 1 to HUNT 2 was associated with a lower prevalence of chronic widespread (OR 0.72, 95% CI 0.56–0.92, Wald = 6.7, p = 0.01), and non-widespread MSCs (OR 0.77, 95% CI 0.68–0.88, Wald = 15.9, p < 0.001) (Table 3). This was not evident for those who decreased their active level from HUNT 1 to HUNT 2 (Table 3). Maintained medium activity level in both surveys was associated with 53% lower prevalence of chronic widespread MSCs (OR 0.47, 95% CI 0.31–0.70, Wald = 13.5, p < 0.001) compared to inactive persons (Table 3).

**Table 3:** Prevalence OR^# ^of chronic musculoskeletal complaints (subdivided to chronic widespread and non-widespread MSCs) related to level of physical activity in HUNT 1 and HUNT 2

Activity level	Total	Chronic MSCs		Chronic widespread MSCs		Chronic non-widespread MSCs	
	39520	n	OR (CI)	n	OR (CI)	n	OR (CI)
**Hunt 1-Hunt 2**							
Inactive-inactive	2058	1165	1.0 (ref.)	152	1.0 (ref.)	1013	1.0 (ref.)
Very low-very low	23671	12147	0.9 (0.8–0.9)	1507	0.8 (0.7–0.9)	10640	0.9 (0.8–1.0)
Low-low	2701	1285	0.9 (0.8–0.9)	165	0.8 (0.6–1.0)	1120	0.9 (0.8–1.0)
Medium-medium	925	385	0.8 (0.7–0.9)	31	0.5 (0.3–0.7)	354	0.8 (0.7–1.1)
High-high	558	229	0.7 (0.6–0.9)	36	0.9 (0.6–1.3)	193	0.7 (0.6–0.9)
Increased level	2342	1089	0.8 (0.7–0.9)	142	0.7 (0.6–0.9)	947	0.8 (0.7–0.9)
Decreased level	2227	1249	0.8 (0.7–0.9)	140	0.8 (0.6–1.1)	1109	1.0 (0.9–1.1)
Missing	5038	2674	0.8 (0.7–0.9)	145	0.4 (0.3–0.5)	2529	0.8 (0.7–0.9)

^#^Adjusted for age, gender, body mass index, smoking and education.

## Discussion

In this large-scale population-based study, individuals who exercised at baseline were less likely to report chronic MSCs, in particular chronic widespread MSCs, 11 years later. Furthermore, consistent medium activity level in HUNT 1 and HUNT 2 was associated with more than 50% lower prevalence of chronic widespread MSCs.

Our findings are in accordance with several cross-sectional [16-18] and prospective [20,24] studies reporting a positive correlation between physical inactivity and MSCs. In contrast, a Dutch population-based study reported that there was no strong correlation between physical activity and MSCs [34], and among 9413 adolescents in Denmark, no association was found [19]. Previous prospective studies have focused on selected groups [20,24]. Our study distinguishes itself from most previous studies by the fact that it focuses on chronic MSCs in a large population study.

Chronic widespread MSCs are associated with an increased risk of cancer and higher mortality [7,8], and to emphasize this important group, we subdivided individuals with chronic MSCs into non-widespread and widespread MSCs. In the present study the impact of physical inactivity on MSCs was most evident for chronic widespread MSCs. Compared to other known risk factors, such as low socio-economic status [6], physical inactivity does not seem to stand out as a very strong risk factor for chronic MSCs. However, as more than 50% of the population suffers from chronic MSCs, a change in the level of physical exercise in the population could potentially have a huge impact on the absolute number of individuals with chronic MSCs. Thus, public health initiatives that increase the level of physical activity in the population may reduce the prevalence of chronic MSCs, in particular chronic widespread MSCs. An increased exercise level in the population may have other positive effects on public health, since physical inactivity is also associated with an increased risk of cancer and higher mortality [12-14].

In the present study, no questions concerning MSCs were included in HUNT 1, and we could therefore not define a population without MSCs at baseline. For this reason, we could not rule out the possibility that MSCs have interfered with the opportunity to achieve or retain a higher physical activity level at baseline. This is in accordance with the fear-avoidance model, whereby fear of pain and associated avoidance behaviour may be an important contributor to developing chronic MSCs [35].

The interval between HUNT 1 and HUNT 2 was 11 years. Despite this long period, the majority of participants reported the same level of physical exercise in the two surveys. Consistent medium activity level in HUNT 1 and HUNT 2 was associated with the most prominent decrease in the prevalence of chronic widespread MSCs. An increased exercise level tended to be more favourable than a decreased level, being associated with a lower prevalence of both chronic widespread and non-widespread MSCs. This was not evident for a decreased activity level. The interpretation of physical activity data in HUNT 2 had to be evaluated with caution, because questions concerning physical activity and chronic MSCs were answered at the same time, and because the level of physical activity was measured somewhat differently in HUNT 2 compared to HUNT 1. Although the combined data supported the positive influence of physical exercise on chronic MSCs, future studies should try to clarify whether chronic MSCs are a cause or a consequence of inactivity.

Strengths of this study include the population-based design with high participation rates, the availability of ample information on several important confounders, the follow-up design, as well as the use of validated questions regarding MSCs and physical activity [27-32]. Since neither musculoskeletal symptoms nor physical activity were the primary objective of the study, interest-related bias appears unlikely. The large sample size decreased the risk of chance findings and facilitated extensive subgroup analyses. The wide range of exposure data made it possible to adjust for potential confounding variables.

Although the attendance rate was high, we can not rule out the possibility of selection bias. Individuals who responded to the musculoskeletal questions were younger, more likely to be women, and had higher socioeconomic status than the non-responders [25,26]. Thus, generalization of our results to those who did not participate should be made with caution.

Age and lower educational levels are risk factors for chronic MSCs [6], but we adjusted for these factors in the final analyses, and potential confounding was also evaluated for other life style factors such as overweight and smoking [3,4,10]. However, there might be other life style factors, such as diet or incompletely registered aspects, which could influence our findings. For example, among those with chronic MSCs, anxiety and other psychological factors may contribute to the experience of pain [36]. It can not be ruled out that anxiety can worsen the symptoms of MSCs and vice versa.

In the present study, a participant's physical activity level is based on leisure time exercise only. However, the impact of occupational physical workload might also have contributed to the results [29]. The term "exercise" was used in all of the three questions regarding physical activity in the HUNT 1 study (Table 1). However, the neutrality of this term must be questioned. For instance, hunting and berry or mushroom picking are particularly common in the county of Nord-Trøndelag. Although these activities involve a certain amount of physical labour, they might not necessarily be considered exercise by the participants of HUNT [28]. Nevertheless, the physical exercise questionnaire in HUNT 1 has been validated recently, and been found to be reproducible and to provide a useful measure of leisure-time physical exercise [27].

## Conclusion

Previous studies evaluating the association between chronic MSCs and physical exercise have shown conflicting results. In this large-scale population-based follow-up study, physical inactivity was associated with a higher prevalence of chronic MSCs, in particular chronic widespread MSCs, supporting the results from two previous prospective studies focusing on more selected groups. This finding may lead to a better understanding of the process leading to this condition. Future studies should try to clarify whether chronic MSCs are a cause or a consequence of inactivity.

## Competing interests

The authors declare that they have no competing interests.

## Authors' contributions

KH, HSH, and HKBW conceived of the study and performed the statistical analysis. KH, HSH, HKBW, and JAZ all participated in the design and drafted the manuscript. HSH and HKBW contributed equally to this work. All authors read and approved the final manuscript.

## Pre-publication history

The pre-publication history for this paper can be accessed here:



## References

[B1] WHO Scientific Group on the Burden of musculoskeletal conditions at the start of the new millenium (2003). The burden of musculoskeletal conditions at the start of the new millennium. Technical Report Series.

[B2] Statistics of sick leave in Norway in 2007. http://www.nav.no.

[B3] Palmer KT, Syddall H, Cooper C, Coggon D (2003). Smoking and musculoskeletal disorders: findings from a British national survey. Ann Rheum Dis.

[B4] Andersson H, Ejlertsson G, Leden I (1998). Widespread musculoskeletal chronic pain associated with smoking. An epidemiological study in a general rural population. Scand J Rehabil Med.

[B5] Anandacoomarasamy A, Caterson I, Sambrook P, Fransen M, March L (2007). The impact of obesity on the musculoskeletal system. Int J Obes.

[B6] Hagen K, Zwart JA, Svebak S, Bovim G, Stovner LJ (2005). Low socio-economic status is associated with musculoskeletal symptoms among 46,901 adults in Norway. Scan J Public Health.

[B7] McBeth J, Silman AJ, Macfarlane GJ (2003). Association of widespread body pain with an increased risk of cancer and reduced cancer survival: a prospective, population-based study. Arthritis Rheum.

[B8] Andersson HI (2004). The course of non-malignant chronic pain: a 12-year follow-up of a cohort from the general population. Eur J Pain.

[B9] Proper KI, Heuvel SG van den, De Vroome EM, Hildebrandt VH, Beek AJ Van der (2006). Dose-response relation between physical activity and sick leave. Br J Sports Med.

[B10] Droyvold WB, Holmen J, Midthjell K, Lydersen S (2004). BMI change and leisure time physical activity (LTPA): an 11-y follow-up study in apparently healthy men aged 20–69 y with normal weight at baseline. Int J Obes.

[B11] Marshall SJ, Jones DA, Ainsworth BE, Reis JP, Levy SS, Macera CA (2007). Race/ethnicity, social class, and leisure-time physical inactivity. Med Sc Sports Exers.

[B12] Nilsen TI, Romundstad PR, Petersen H, Gunnell D, Vatten LJ (2008). Recreational physical activity and cancer risk in subsites of the colon (the Nord-Trøndelag health study). Cancer Epidemiol Biomarkers Prev.

[B13] Ellekjaer H, Holmen J, Ellekjaer E, Vatten L (2000). Physical activity and stroke mortality in women: Ten-year follow-up of the Nord-Trondelag health survey, 1984–1986. Stroke.

[B14] Warburton DER, Nicol CW, Bredin SSD (2006). Health benefits of physical activity: the evidence. CMAJ.

[B15] Hildebrandt VH, Bongers PM, Dul J, van Dijk FJH, Kemper HCG (2000). The relationship between leisure time, physical activities and musculoskeletal symptoms and disability in worker populations. Int Arch Occup Environ Health.

[B16] Morken T, Mageroy N, Moen BE (2007). Physical activity is associated with a low prevalence of musculoskeletal disorders in the Royal Norwegian Navy: a cross sectional study. BMC Musculoskelet Disord.

[B17] Nabeel I, Baker BA, McGrail MPj, Flottemesch TJ (2007). Correlation between physical activity, fitness and musculoskeletal injuries in police officers. Minn Med.

[B18] Simes E, Sødal E, Nurk E, Tell GS (2003). Epidemiology of musculoskeletal complaints in Hordaland, Norway. Tidsskr Nor Lægeforen.

[B19] Andersen LB, Wedderkopp N, Leboeuf-Yde C (2006). Association between back pain and physical fitness in adolescents. Spine.

[B20] Bruce B, Fries JF, Lubeck DP (2005). Aerobic exercise and its impact on musculoskeletal pain in older adults: a 14 year prospective, longitudinal study. Arthritis Res Ther.

[B21] Mikkelsson M, El-Metwally A, Kautiainen H, Auvinen A, Macfarlane GJ, Salminen JJ (2008). Onset, prognosis and risk factors for widespread pain in schoolchildren: a prospective 4-year follow-up study. Pain.

[B22] Malchaire J, Cock N, Vergracht S (2001). Review of the factors associated with musculoskeletal problems in epidemiological studies. Int Arch Occup Environ Health.

[B23] Verbunt VA, Huijnen IPJ, Köke A Assessment of physical activity in daily life in patients with musculoskeletal pain. Eur J Pain.

[B24] Miranda H, Viikari-Juntura E, Martikainen R, Takala EP, Riihimäki H (2001). A prospective study of work related factors and physical exercise as predictors of shoulder pain. Occup Environ Med.

[B25] Holmen J, Midthjell K, Bjartveit K, Hjort PF, Lund-Larsen PG, Moum T (1990). The Nord-Trøndelag Health Survey 1984–86: Purpose, background and methods. Participation, non-participation and frequency distribution.

[B26] Holmen J, Midthjell K, Krüger Ø, Langhammer A, Holmen TL, Bratberg GH, Vatten L, Lund-Larsen PG (2003). The Nord-Trøndelag Health Study 1995–1997 (HUNT 2): Objectives, contents methods and participation. Nor J Epidemiol.

[B27] Kurtze N, Rangul V, Hustvedt BE, Flanders WD (2008). Reliablility and validity of self-reported physical activity in the Nord-Trøndelag Health Study HUNT 1. Scand J Public Health.

[B28] Kurtze N, Gundersen KT, Holmen J (2003). Self-reported physical activity in population studies – a methodological problem. Nor J Epidemiol.

[B29] Kurtze N, Rangul V, Hustvedt BE, Flanders WD (2007). Reliability and validity of self-reported physical activity in the Nord-Trondelag Health Study (HUNT 2). Eur J Epidemiol.

[B30] Kuorinka I, Jonsson B, Kilbom A, Vinterberg H, Biering-Sørensen F, Andersson G, Jørgensen K (1987). Standardised Nordic Questionnaires for the analysis of musculoskeletal symptoms. Appl Ergon.

[B31] Franzblau A, Salerno DF, Armstrong TJ, Werner RA (1997). Test-retest reliability of an upper-extremity discomfort questionnaire in an industrial population. Scand J Work Environ Health.

[B32] Palmer K, Smith G, Kellingray S, Cooper C (1999). Repeatability and validity of an upper limb and neck discomfort questionnaire: The utility of the Standardized Nordic Questionnaire. Occup Med.

[B33] Skoffer B, Foldspang A (2008). Physical activity and low-back pain in schoolchildren. Eur Spine J.

[B34] Wijnhoven HAH, de Vet HCW, Picavet HSJ (2006). Explaining sex differences in chronic musculoskeletal pain in a general population. Pain.

[B35] Leeuw M, Goossens MEJB, Linton SJ, Crombez G, Boersma K, Vlaeyen JWS (2007). The Fear-Avoidance Model of Musculoskeletal Pain: Current State of Scientific Evidence. J Behav Med.

[B36] Benjamin S, Morris S, McBeth J, Macfarlane GJ, Silman AJ (2000). The association between chronic widespread pain and mental disorder: A population-based study. Arthritis Rheum.

